# Optimization of In Vitro Shoot Culture Parameters for Enhanced Biomass and Rosmarinic Acid Production in *Salvia atropatana*

**DOI:** 10.3390/molecules30122654

**Published:** 2025-06-19

**Authors:** Wiktoria Ejsmont, Anna K. Kiss, Izabela Grzegorczyk-Karolak

**Affiliations:** 1 Department of Biology and Pharmaceutical Botany, Medical University of Lodz, 90-151 Lodz, Poland; wiktoria.ejsmont@student.umed.lodz.pl; 2 Department of Pharmaceutical Biology, Medical University of Warsaw, 02-097 Warsaw, Poland; anna.kiss@wum.edu.pl

**Keywords:** cytokinins, harvest period, plant growth regulators, polyphenolic compounds, shoot proliferation, TOPSIS

## Abstract

*Salvia atropatana* is a medicinal plant native to Middle Eastern countries. It has been traditionally used in Turkish and Iranian folk medicine to treat infections, wounds, inflammatory diseases, spastic conditions, and diabetes. Its therapeutic potential has been attributed to its essential oil, polyphenolic acid, flavonoid, and diterpenoid content. The aim of the study was to determine the optimal conditions of in vitro *S. atropatana* shoot culture to enhance proliferation and secondary metabolite production. It examined the effects of various cytokinins and culture duration on culture growth parameters and phenolic compound accumulation. Exogenous cytokinin supplementation significantly enhanced shoot proliferation, with the highest proliferation ratio (6.3) observed with 1 and 2 mg/L 6-benzylaminopurine (BAP). Biomass accumulation was the highest at 0.5 mg/L BAP, followed by 1 and 2 mg/L meta-toplin (mTOP). Phenolic profiling identified nine compounds, with rosmarinic acid (RA) as the dominant metabolite. The highest RA content (16 mg/g dry weight) was achieved with 1 and 2 mg/L BAP and 0.5 mg/L of its ryboside. The TOPSIS (Technique for Order of Preference by Similarity to Ideal Solution) method identified 1 mg/L BAP as the optimal treatment, balancing high proliferation, biomass, and polyphenol accumulation. Extending culture duration to 50 days increased biomass and phenolic content reaching 19.25 mg/g dry weight. However, morphological changes, including apical necrosis, were observed, and a significantly longer cultivation period was needed, questioning the value of the procedure. This study provides a basis for scalable in vitro production of bioactive compounds in *S. atropatana*.

## 1. Introduction

Modern research on plant-derived medicines is increasingly focused on the treatment and prevention of civilization-associated diseases, and has yielded promising results. One prominent example is rosmarinic acid (RA), a compound renowned for its anti-inflammatory and antioxidant properties. RA demonstrates significant cardioprotective and hepatoprotective effects and serves as a neuroprotective agent with the potential to inhibit the aggregation and formation of Tau filaments associated with Alzheimer’s disease. Additionally, RA exhibits notable anticancer activity [1,2]. Despite the growing body of evidence supporting the therapeutic potential of RA and its derivatives, the development of high-quality, standardized preparations remains a challenge due to limited availability and rising costs.

A significant commercial source of RA is rosemary (*Rosmarinus officinalis* L.) [3]. However, its secondary metabolite production is strictly dependent on external factors, such as the time and location of harvesting, chemotypes, and variations within the same genus [4,5]. The amount of RA in plant material is also associated with the harvest time. For example, in rosemary plants grown in southwest Romania, foliar RA content reaches a maximum in April, and minimum in August [6]. In contrast, biotechnological approaches enable the production of genetically stable cultures that yield reproducible quantities of secondary metabolites, thus ensuring consistent quality and lower raw material costs; plant production is also independent of environmental variability, which is an additional advantage in the face of ongoing climate change [7].

Within the Lamiaceae family, the genus *Salvia* has garnered substantial attention due to its high concentrations of polyphenolic compounds [8,9]. Various *Salvia* species are well documented for their aromatic, antioxidant, antimicrobial, and anticancer properties [10], and clinical trials have been found to be effective in addressing neurodegenerative disorders and as cardioprotective agents [11,12].

Among the lesser-studied members of this genus, *Salvia atropatana* stands out. It is a medicinal plant native to Iran, Iraq, and Turkey, and is traditionally used in Iranian folk medicine to treat infections, wounds, inflammatory diseases, spastic conditions, and diabetes. Its therapeutic potential is attributed to the presence of diverse secondary metabolites, including essential oils, di- and triterpenoids, polyphenolic acids, and flavonoids [13,14,15,16,17,18]. Rosmarinic acid was detected in the leaves and roots of plants collected from the wild in northern Iran at levels of 6.55 mg/g and 1.65 mg/g dry weight, respectively [17]. However, biotechnological studies on *S. atropatana* are limited to a single report on callus culture, which identified it as a promising source of RA and other polyphenolic acids [19].

In contrast, optimized shoot cultures have been described for other sage species with high yields. Shoot cultures of *S. bulleyana* Diels grown under optimal light conditions achieved twice the total phenol content then noted in the above-ground part of two-year-old intact plants [20]. A culture of *S. virgata* Jacq. shoots elicited by Ag^+^ ions yielded high RA and caffeic acid contents [21]; in addition, *S. viridis* L. shoot culture achieved significantly greater culture growth and phenolic compound content when cytokinin type and concentration in the growth medium was optimized [22].

The addition of exogenous growth regulators, and the optimization of their concentrations in the medium, is a widely employed and effective strategy for enhancing secondary metabolite production and biomass in in vitro plant cultures. Plant growth regulators affect cell division, metabolic processes, and plant growth [23]. Among these, exogenous cytokinins play important roles in regulating cell division, their differentiation, shoot proliferation, and organogenesis, and are essential for micropropagation [24,25]. Their concentrations, and their ratio with auxins, determine morphogenetic outcomes: higher cytokinin-to-auxin ratios promote shoot formation, while lower ratios induce root development. However, some evidence suggests that the classical model of cytokinin-to-auxin ratio determining organogenic direction has been based on complex, multifactorial hormone interactions and regulatory mechanisms. Recent reports suggest that exogenous cytokinins exert their primary influence on shoot induction through the stimulation and maintenance of endogenous auxin biosynthesis [26]. Moreover, excessive exogenous cytokinin levels can lead to hyperhydricity, and abnormal shoot morphology; as such, it is necessary to precisely optimize exposure according to specific plant species and culture [24,25].

The most widely used cytokinin in plant in vitro cultures, including sage (*Salvia* spp.) propagation, is benzylaminopurine (BAP), due to its strong ability to induce shoot proliferation, axillary bud activation, and adventitious shoot formation [27,28,29]. However, BAP analogs including glycosylated and hydroxylated derivatives have recently been tested for optimizing plant tissue culture efficiency and enhancing bioactive compound biosynthesis. Some studies have indicated that they offer improved stability and specificity, and reduced side effects such as hyperhydricity, while maintaining high morphogenic activity [22,30,31].

In the present study, the effects of different cytokinin types on the growth of shoot culture of sage (*S. atropatana*) and the production of secondary metabolites, including rosmarinic acid, were evaluated. The tested cytokines included BAP, rBAP (6-benzylaminopurine riboside), BPA (N-benzyl-9-(tetrahydropyranyl)-adenine), and mTOP (meta-topolin) at concentrations of 0.5, 1.0, and 2.0 mg/L. The optimal harvest time was determined by analyzing plant material at three distinct intervals (30, 40, and 50 days). This report is the first such comprehensive study of *S. atropatana* shoot culture optimization for secondary metabolite production.

## 2. Results

### 2.1. Effect of Exogenous Cytokinins

#### 2.1.1. Culture Growth

The shoot culture used in this experiment was maintained on MS agar medium supplemented with 0.1 mg/L IAA (indole-3-acetic acid) and 1 mg/L mTOP and subcultured onto fresh growth medium every five weeks. The shoots utilized in the experiment were obtained from the twelfth subculture (approximately one year of culture).

The addition of cytokinin to the medium, regardless of its type and concentration, stimulated shoot proliferation (Table 1). In the presence of cytokinin, 93–100% of explants regenerated shoots, whereas for shoots grown on medium with auxin alone (control), this was 25%.

**Figure 1:**
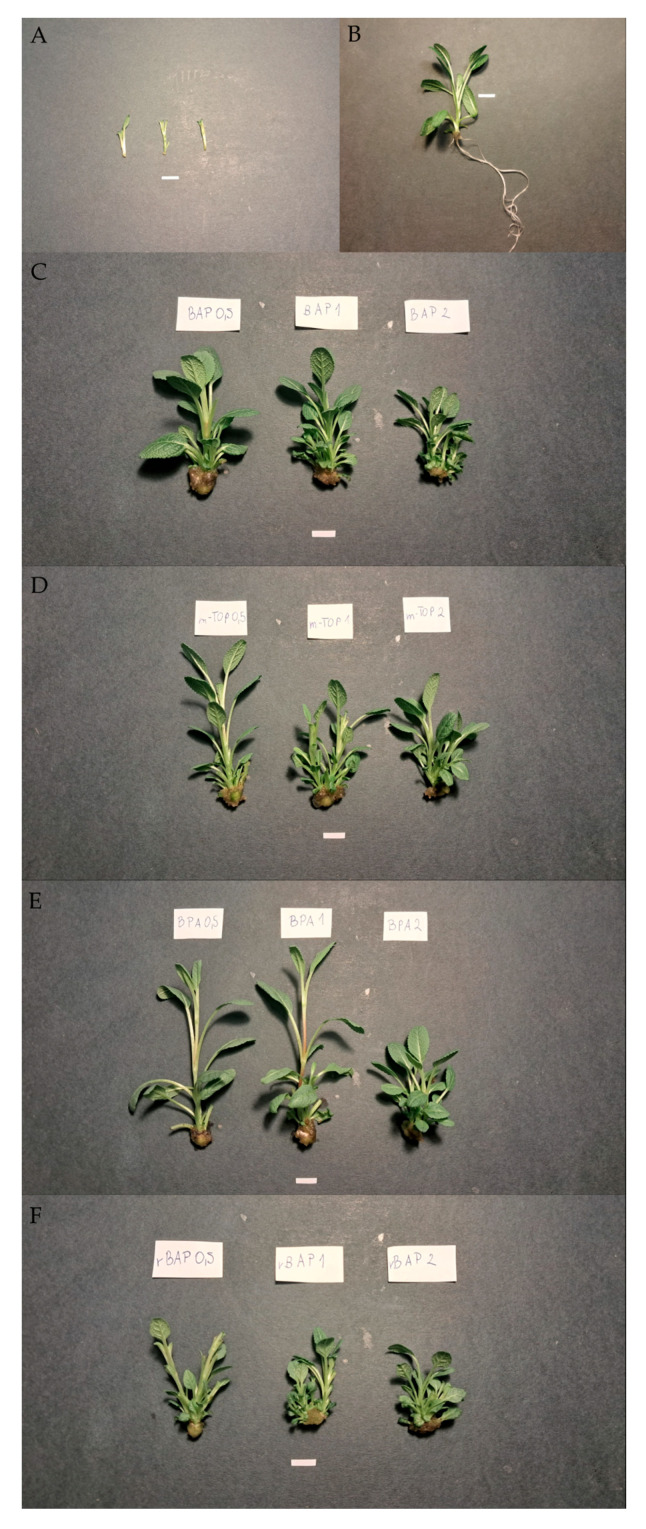
*S. atropatana* shoot culture. Explant used in experiment (**A**); shoot cultivated on medium with 0.1 mg/L IAA (**B**); shoots cultivated on media supplemented with 0.1 mg/L IAA: and BAP (**C**), and mTOP (**D**), and BPA (**E**), and rBAP (**F**). Scale bar = 1 cm.

**Figure 2:**
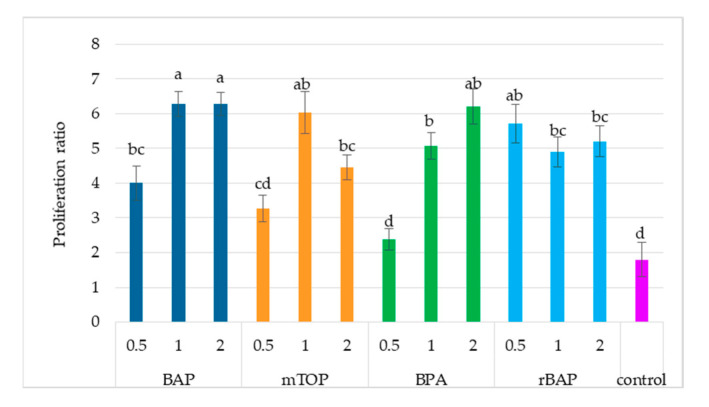
The proliferation ratio of *S. atropatana* culture with regard to the type and concentration of applied cytokinins. Means sharing the same letter did not share significant differences at *p* < 0.05.

All types and concentrations of cytokinins tested, except the lowest concentration of BPA, increased the proliferation ratio of *S. atropatana* cultures (Figure 1 and Figure 2). The highest proliferation ratio was achieved with BAP at concentrations of 1 mg/L and 2 mg/L (proliferation ratio: 6.3), which was over three times higher than control values (1.8). The addition of 1 mg/L mTOP, 2 mg/L BPA, and 0.5 mg/L rBAP was similarly beneficial for proliferation, with no statistically significant differences observed among these treatments (proliferation ratio values: 5.7–6.2) (Figure 1).

The lowest concentration of cytokinin in the medium resulted in a higher percentage of obtained shoots compared to buds (55–63%, depending on the cytokinin applied) (Table 1). The best results were obtained in the presence of 0.5 mg/L cytokinin, with 63.13% of shoots and 36.88% of buds. As the cytokinin concentration in the medium increased, this percentage decreased for all BAP derivatives, reaching 47–50% at a concentration of 2 mg/L. An exception was unsubstituted BAP, where a concentration of 2 mg/L still resulted in an unchanged predominance of shoots over buds (57%).

**Figure 3:**
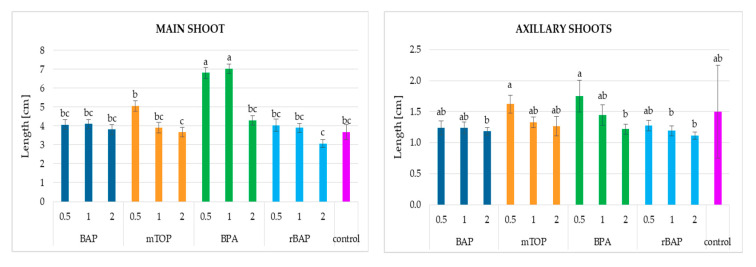
The height of *S. atropatana* main and axillary shoots with regard to the type and concentration of applied cytokinin. Means sharing the same letter did not share significant differences at *p* < 0.05.

The mean length of the main shoot obtained from the explant for most treatments ranged between 4 and 5 cm (Figure 3). An exception was observed for shoots growing on the substrate with 2 mg/L rBAP, which only reached 3 cm. In contrast, significantly longer main shoots were observed for treatments with 0.5 and 1 mg/L BPA, reaching approximately 7 cm.

The longest axillary shoots were obtained with the lowest concentrations of BPA and mTOP (above 1.6 cm) (Figure 3). However, only a few instances demonstrated statistically significant differences in shoot length. In addition, both the main and axillary shoots tended to be shorter at higher cytokinin concentrations.

**Figure 4:**
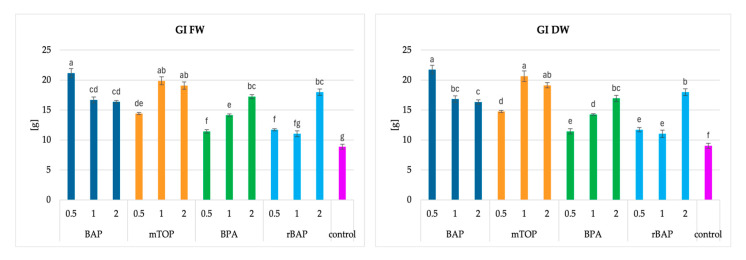
The growth index (fresh and dry weight—FW, DW) of *S. atropatana* culture with regard to the type and concentration of applied cytokinins. Means sharing the same letter did not share significant differences at *p* < 0.05.

The most important growth parameter for high productivity is biomass accumulation. The highest growth indices (GI) for fresh weight (FW) and dry weight (DW) were observed in shoots cultivated in the presence of 0.5 mg/L BAP, reaching 21.2 FW and 21.7 DW (Figure 4). Similarly, notable results were recorded for mTOP at concentrations of 1 and 2 mg/L, with GIs of approximately 20. These values were more than twice those obtained for the control group.

Relatively low growth indices were noted at lower rBAP levels and the lowest BPA concentration. In the former, this could be attributed to the development of relatively small leaves on shoots grown in the presence of ribosylated BAP. In the latter, the primary reason was the low proliferation ratio of 0.5 mg/L BPA-treated culture. For both of these cytokinins, the best results were achieved at their highest concentrations, whereas BAP displayed the opposite trend (Figure 4).

#### 2.1.2. Secondary Metabolite Accumulation

**Figure 5:**
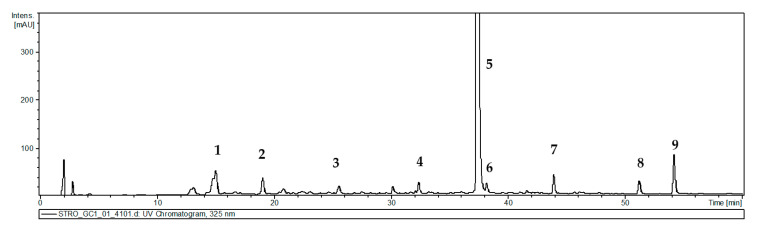
UV chromatogram of *S. atropatana* 80% methanolic shoot extract. Peak numbers refer to those used in Table 2.

Nine phenolic compounds were detected in the hydromethanolic extract from *S. atropatana* shoots (Table 2, Figure 5). These included rosmarinic acid (RA), its hexoside (RAH) and methyl ester (MRA), caffeic acid (CA), its derivative: caffeoyl-threonic acid (CTA), prolithospermic acid (PLA), salvianolic acid K (SAK), and two isomers of salvianolic acid F (SAF I and SAF II). All these compounds were earlier reported in other *Salvia* species, both in the above-ground parts of field-grown plants [32,33,34] and in shoots cultivated in vitro [35].

**Figure 6:**
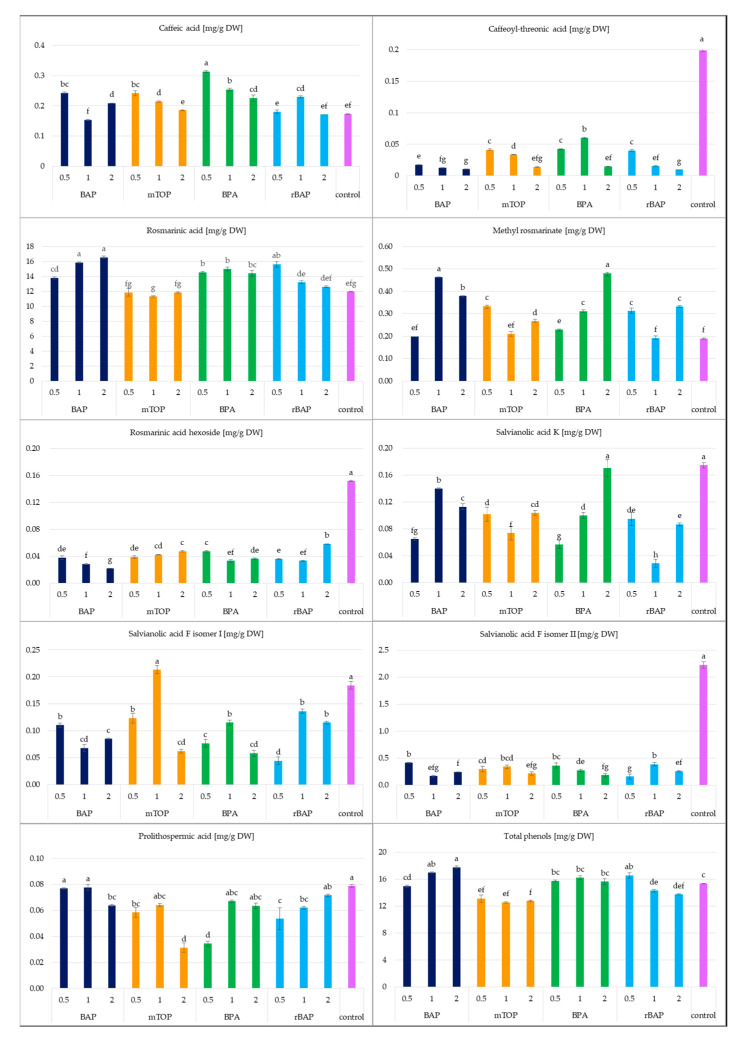
The polyphenol content in the *S. atropatana* culture with regard to the type and concentration of applied cytokinins. Means sharing the same letter did not share significant differences at *p* < 0.05.

The dominant phenolic acid in plant material for all treatments was RA, accounting for over 90% of the total phenolic content in cultures grown on medium supplemented with cytokinins (Figure 6). Its share was lower in control shoots, constituting approximately 70% of the quantified polyphenols.

The highest RA content (~16 mg/g DW) was observed in shoots cultured on medium with 1 and 2 mg/L BAP and 0.5 mg/L rBAP, representing a 30% increase compared to the control (Figure 6). Conversely, supplementation with mTOP at all tested concentrations resulted in RA levels similar to those in the control, indicating that mTOP was the least effective cytokinin for RA biosynthesis in *S. atropatana* cultures.

Other metabolites were present in significantly smaller amounts (<0.5 mg/g DW), with treatment efficacy varying for different compounds. Notably, the concentrations of SAF II, CTA, and RAH were significantly higher in shoots cultured on MS medium supplemented with auxin alone compared to those grown with cytokinins.

The highest total polyphenol content (17.7 mg/g DW) was achieved in cultures grown on medium with 2 mg/L BAP, followed by those with 1 mg/L BAP and 0.5 mg/L rBAP (without statistical significance).

Due to the differences noted in the responses of individual growth parameters and the biosynthesis of polyphenolic compounds, it was not possible to clearly identify an optimal cytokinin combination for polyphenolic compound accumulation in *S. atropatana* shoot cultures. Therefore, a TOPSIS analysis (Technique for Order of Preference by Similarity to Ideal Solution) was employed (Table 3), a method previously demonstrated to be effective in optimizing in vitro production [34].

The decision-making process included four parameters deemed most critical: rosmarinic acid content, total phenolic acid content, growth index based on dry weight (DW), and proliferation ratio; each was assigned an equal weight of 0.25. Based on the analysis, the highest performance score was observed for *S. atropatana* shoots cultivated on a medium supplemented with 1 mg/L BAP (0.782), followed by 2 mg/L BAP (0.773). These treatments exhibited the highest proliferation ratio, moderate biomass accumulation, and the greatest accumulation of rosmarinic acid and total polyphenol content in the culture.

Finally, the treatment with 1 mg/L BAP was identified as the optimal choice, as it not only achieved the highest performance score but also offered cost advantages, as it required lower cytokinin levels compared to 2 mg/L BAP.

### 2.2. Effect of the Culture Duration

#### 2.2.1. Culture Growth

**Figure 7:**
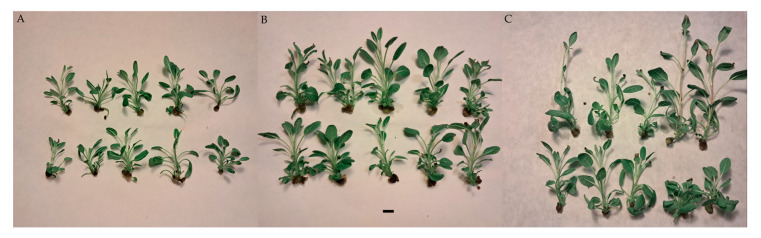
*S. atropatana* shoot culture after 30-day cultivation (**A**) 40-day cultivation (**B**), 50-day cultivation (**C**) on MS medium supplemented with 0.1 mg/L IAA and 1 mg/L BAP. Scale bar = 1 cm.

In the next step, *S. atropatana* shoots cultured on a medium containing a previously selected combination of growth regulators (0.1 mg/L IAA and 1 mg/L BAP) were evaluated to determine the optimal harvest time for rosmarinic acid production. For this purpose, samples were collected every 10 days, from day 30 to day 50 of cultivation (Figure 7).

**Figure 8:**
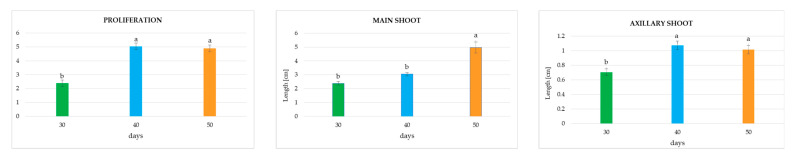
The proliferation ratio and height of *S. atropatana* shoots with regard to harvest time. Means sharing the same letter did not share significant differences at *p* < 0.05.

**Figure 9:**
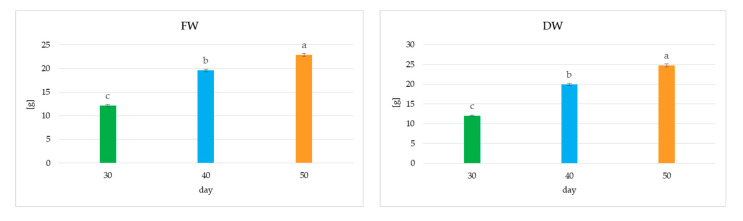
The growth index (fresh and dry weight—FW, DW) of *S. atropatana* culture with regard to harvest time. Means sharing the same letter did not share significant differences at *p* < 0.05.

The proliferation of *S. atopatana* culture increased significantly until day 40, reaching a mean value of five shoots/buds per explant, after which it stabilized (Figure 8). In contrast, biomass continued to increase over the next 10 days by approximately 15–20%, while the main shoot elongated by nearly 70% (Figure 8 and Figure 9). However, after 50 days of cultivation, the culture conditions deteriorated markedly, with drying of the shoot tips and the leaf tips observed (Figure 7C).

#### 2.2.2. Secondary Metabolite Accumulation

**Figure 10:**
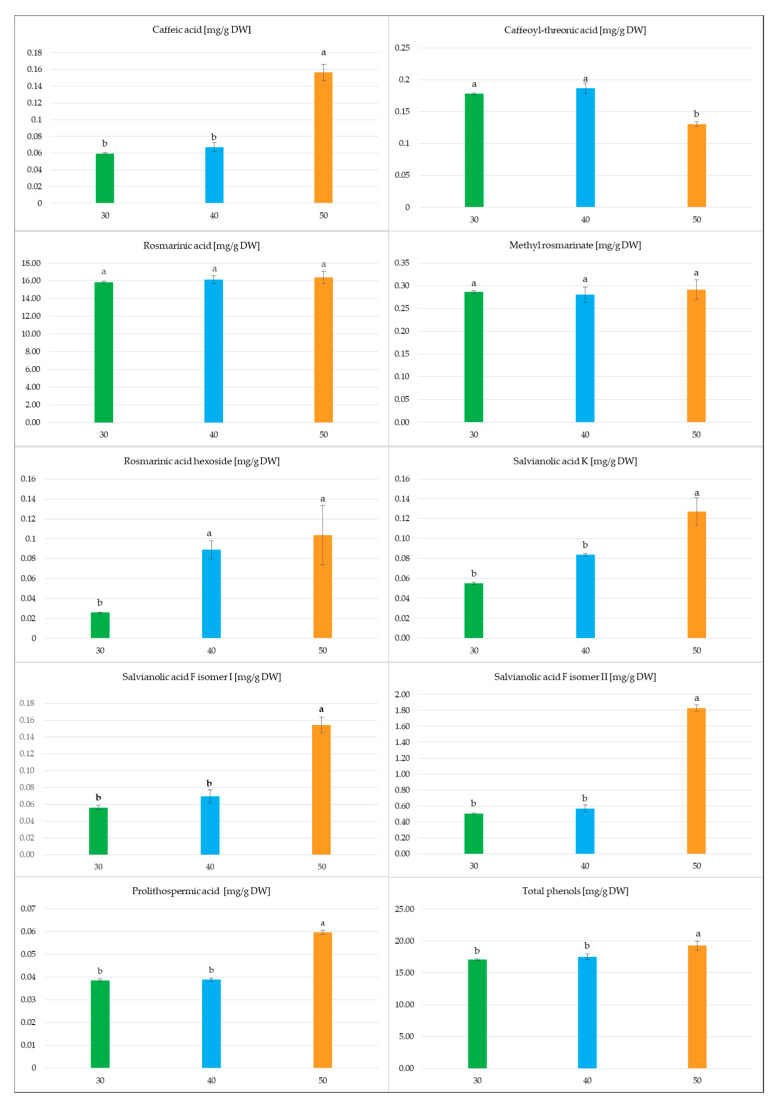
The polyphenol content in the *S. atropatana* culture with regard to harvest time. Means sharing the same letter did not share significant differences at *p* < 0.05.

The length of cultivation influenced the metabolite content in *S. atropatana* culture (Figure 10). In most cases, it increased over time, doubling between days 30 and 50 for PLA, SAFI, SAK, and CA, or even tripling or more for SAF II and RAH. However, the opposite effect was observed for CTA. In contrast, the levels of the main metabolites in this culture, RA and its methyl ester, did not change significantly between day 30 and 50, reaching 15.8–16.4 mg/g DW and 0.28–0.29 mg/g DW, respectively. Ultimately, between days 30 and 40, the total polyphenol level increased by 3%, followed by an additional 10% increase over the next 10 days, reaching a maximum of 19.25 mg/g DW (Figure 10). The concurrent increase in dry matter between days 40 and 50 suggests that extending the cultivation period may be beneficial for achieving higher productivity; however, while an almost doubling increase in biomass was noted between days 30 and 40, the gain observed from day 40 to 50 was less notable. As such, further evaluation is needed to determine whether it is significant enough to justify the additional costs associated with extending the cultivation period.

## 3. Discussion

Traditional cultivation of medicinal plants is often hindered by environmental factors such as climate variability, soil conditions, and susceptibility to pests and diseases, leading to inconsistent yields of bioactive compounds. In vitro plant tissue culture offers a viable alternative, providing a controlled environment that is independent of external conditions, thereby ensuring stable and continuous production of these valuable metabolites [8]. In vitro culture also offers the significant advantage of being able to manipulate the culture medium to enhance both growth and secondary metabolite biosynthesis. Modifying the composition of the culture medium or changing the types and concentrations of cytokinins in the medium can significantly influence the production of polyphenolic compounds in in vitro plant cultures. For example, shoot proliferation of *Scutellaria altissima* L. and *S. alpina* L. was most effective with 2–4 µM BAP, achieving 5.7–6.4 and 23.5–25 shoots per explant, respectively, over five weeks. Alternatively, tidiazuron (TDZ) treatment at 1 µM for *S. altissima* and 0.5 µM for *S. alpina* was found to be optimal for the biosynthesis of flavones, including baicalin and wogonoside. Under the same conditions, verbascoside accumulation also increased [36].

Cytokinin type and concentration were also found to influence the rosmarinic acid (RA) content in *Thymus leucotrichus* Halácsy [37]. The highest RA content (10.15 mg/g DW) was achieved with 0.5 mg/L 2-isopentenyladenine (2iP). Lower concentrations of 2iP also promoted higher RA production, whereas higher concentrations were less effective. In contrast, BAP supplementation negatively impacted RA production in *Thymus* culture, reducing its levels below those found in natural sources and cytokinin-free cultures [37]. The differential impact of BAP on polyphenolic metabolism was further evident in *Dracocephalum forrestii* W.W. Smith: while 0.5 mg/L BAP enhanced the accumulation of both RA and salvianolic acid B, it failed to support shoot proliferation or biomass accumulation [38]. Increasing BAP concentration to 2 and 4 mg/L improved growth parameters more than twofold; however, this increase came at the cost of a dramatic, i.e., up to threefold, reduction in polyphenolic acid levels.

Similar cytokinin-dependent trends have been observed in *Salvia* spp. cultures, where BAP seldom yields optimal polyphenol accumulation, despite being frequently employed due to its availability and cost-effectiveness [27,39,40,41]. For instance, 1.5 mg/L BAP was optimal for shoot proliferation in *Salvia officinalis* L., but kinetin at the same concentration enhanced RA content by more than 50% compared to BAP-treated explants [42]. On the other hand, BAP was ineffective for both growth and metabolite production in two other *Salvia* species, whereas its substituted derivatives, such as mTOP, rBAP, and BPA, significantly stimulated polyphenol production and, in many cases, also enhanced growth and culture quality. In *S. viridis*, supplementation with 2 mg/L BPA resulted in the highest total polyphenol content, including RA, nearly double that of control cultures, whereas 1 mg/L mTOP significantly enhanced phenylethanoid accumulation, with levels more than three times higher than in control cultures. This last cytokinin also optimized biomass accumulation for *S. viridis* [22]. The maximum RA content in *S. bulleyana* shoot extracts was obtained from cultures grown on medium containing 2 mg/L ribosylated BAP [35]. In contrast, the highest proliferation ratio for *S. bulleyana* was observed on medium supplemented with 2 mg/L BPA, while biomass accumulation was optimized with 1 mg/L mTOP.

The results of these two studies provide a rationale for using three different BAP derivatives in addition to traditional BAP to optimize the growth and production of *S. atropatana* shoots. Ultimately, all of the treatments used positively influenced both growth and metabolite production in this species. However, for *S. atropatana*, the most beneficial treatment turned out to be the supplementation of the growth medium with 1 mg/L BAP, indicative of the high species-specificity of this procedure. The findings also confirm that strategic manipulation of cytokinin types and their concentrations in culture media can effectively enhance the biosynthesis of specific polyphenolic compounds, including rosmarinic acid, in sage shoot cultures.

The influence of cytokinin type and concentration on shoot proliferation, biomass production, and secondary metabolite accumulation in vitro is well documented; however, it is realized via complex and insufficiently characterized mechanisms. Structural differences among cytokinins affect their chemical stability, receptor binding affinity, and specificity, leading to variations in signal transduction and genotype-dependent differences in biological responses. Moreover, cytokinin-induced effects are dose-dependent and subject to feedback inhibition and metabolic reprogramming, complicating the interpretation of their regulatory roles. Although cytokinins are recognized modulators of plant growth and specialized metabolism, their direct involvement in RA biosynthesis and the regulation of key enzymes in the phenylpropanoid pathway in *Salvia* spp. remain poorly understood. Nonetheless, evidence suggests that cytokinins can upregulate genes such as *PAL*, which catalyzes the first stage into the phenylpropanoid pathway. For instance, in *Satureja spicigera* (K.Koch) Boiss., exogenous cytokinin application significantly enhanced *PAL* gene expression and RA content in shoots, indicating a correlation between cytokinin-induced signaling, *PAL* activation, and elevated RA biosynthesis [43]. Similarly, in grapevine, exogenous cytokinin treatment resulted in transcriptional upregulation of phenylpropanoid-related genes, accompanied by modifications in polyphenolic metabolite profiles [44]. Further, studies of *Arabidopsis* and other model species suggest that cytokinin regulation of *PAL* activity may occur post-transcriptionally [45].

The next step was to determine the optimal moment for harvesting plant material, as collecting plants at the most appropriate developmental stage is a key factor in maximizing phytochemical yield [46,47]. The studies pointed to the existence of diurnal variations in secondary metabolite production [48]. For instance, oil yield in *Pelargonium* was highest at noon, whereas in *S. officinalis*, peak oil content occurred between 4 and 6 p.m. [48,49]. Also, the biologically active compound content has also been found to vary with the stage of plant development. Reports in the literature indicate that RA levels in the aerial parts of *S. officinalis* and *Heracleum persicum* Desf. ex Fisch. peak during the vegetative stage, and decline gradually in the further growth cycle [47,50]. However, in *Origanum majorana* L., the highest polyphenol content was recorded during the late vegetative phase, whereas essential oil peaked at full flowering [51]. In addition, the highest polyphenol content was reported at different phenological stages depending on species: floral budding in *Hypericum hyssopifolium* Chaix, full flowering in *H. pruinatum* Boiss. & Balansa, and fresh fruiting in *H. nummularioides* Trautv [52]. These findings emphasize that metabolite accumulation patterns are not only species-dependent but also metabolite-specific and reflect the specific physiological roles of the bioactive compounds in plants. For example, the increase in aromatic oil content during flowering serves to increase the attractiveness of flowers to pollinators, whereas polyphenol accumulation is often stimulated by environmental factors [52]. Plant growth and secondary metabolism can also be significantly influenced by stress factors such as drought, temperature fluctuations, salinity, and pathogens, ultimately shaping the phytochemical profile [53,54]. Plants respond to such stressors by synthesizing phenolic compounds as part of their defense mechanism, thus mitigating oxidative stress and ecological interactions.

In vitro culture offers a valuable platform for controlled production of secondary metabolites by minimizing environmental variability. However, even under in vitro conditions, plant cells progress through growth phases that influence metabolite synthesis. This is particularly evident in dynamic systems such as suspension cultures, where the timing of biomass changes and secondary metabolite production have been analyzed in detail. For example, in *Lavandula vera*, DC. biomass and RA levels peaked on day 8 of cultivation, followed by a sharp decline [55]. Similar growth-production kinetics were observed in *Salvia fruticosa* Mill. callus and suspension cultures, with maximum RA content occurring after 35 and 20 days, respectively [56]; in contrast, the highest biomass growth of *Ocimum sanctum* L. culture was observed at the sixth week, together with maximum RA content [57]. In cell cultures of *Anchusa officinalis* L., RA biosynthesis commenced upon the decline of primary metabolism, which coincided with nutrient depletion [58]. In *Lavandula* culture, the biosynthesis of rosmarinic acid also began to increase when the levels of nitrogen compounds and sugar in the medium dropped drastically [55]. These results suggest that stress induced by nutrient depletion, accumulation of toxic compounds, and other limiting factors may act as a trigger for enhanced secondary metabolism in in vitro culture.

The optimization of harvest time is important for both cell culture and organ cultures. Frequently, longer growth periods provide plants with more time to allocate resources to the production of secondary metabolites. It has also been found that the biosynthesis of polyphenols most often correlates with the growth phases, with rosmarinic acid reporting production peaks in the stationary phase after the end of the intensive growth phase. However, the profile of production has been found to be species-specific. For example, hairy roots of *Agastache rugosa* (Fisch. & C.A.Mey.) Kuntze achieved peak biomass and RA content after 14 days [59], and *S. viridis* on day 30 [22]. Notably, longer growth durations do not always correlate with higher phytochemical content due to limitations such as nutrient exhaustion, and onset of senescence: in *Mentha* spp., maximum phenolic content was detected before shoot biomass culmination [60], whereas in *Fagopyrum esculentum* Moench cultures, catechins and procyanidins reached their maximum at day 24, while anthocyanins peaked on day 8, despite optimal growth occurring on day 20 [61].

Therefore, as with field-grown plants, the timing of harvest in in vitro cultures must be strategically optimized to coincide with maximal metabolite accumulation; organ culture optimization also holds promise for cost reduction by shortening the culture period while maintaining product yield. In the case of *S. atropatana* shoots, a 40-day cultivation period appears optimal. Although biomass increases slightly over the subsequent 10 days, the RA content remains stable, rendering further culture extension economically unjustified. In contrast, if the aim were to maximize the accumulation of salvianolic acids such as SAK, SAF isomers, and PLA, it may be advisable to extend the culture to 50 days, as their levels increase significantly between days 40 and 50.

## 4. Materials and Methods

### 4.1. Plant Material

In vitro culture was obtained from *Salvia atropatana* seeds, originating from the Kärntner Botanikzentrum (Klagenfurt am Wörthersee, Austria). Seeds were sterilized as previously mentioned [19] and cultivated on solidified MS (Murashige and Skoog) medium [62] supplemented with 30 g/L sucrose, 0.02 mg/L kinetin, and 1 mg/L gibberellic acid. The shoot tips of the seedlings were cut and transferred to MS medium supplemented with 30 g/L sucrose, 0.1 mg/L IAA, and 1 mg/L mTop. The culture was grown in a growth room at 26 ± 2 °C and photoperiod (16 h/8 h) using white fluorescent lamps (40 µM/m^2^∙s), subculturing every five weeks to fresh medium.

### 4.2. Culture Proliferation, Growth, and Biomass Accumulation

Single shoot apexes of five-week-old plants, cultivated in vitro, were placed in glass tubes, filled with 25 mL of solid (0.7% agar) MS medium, supplemented with 30 g/L sucrose, 0.1 mg/L IAA, and one of four different types of purine-based cytokinin (BAP, rBAP, mTOP, and BPA) at three different concentrations (0.5, 1, and 2 mg/L). The control consisted of shoots cultivated on a MS medium with addition of only auxin (IAA). Regulators were obtained from Duchefa Biochemie B.V. (Haarlem, The Netherlands). The pH of the medium was set at 5.7–5.8. The mean weight of the inoculum was approximately 0.039 g fresh and 0.0038 g dry. Explants were grown under a sixteen-hour photoperiod using white fluorescent lamps (40 µM/m^2^∙s), at 26 ± 2 °C. The experiment was conducted three times; each time it consisted of at least 10 different explants for each treatment (subculture 19–21). After five weeks, the percentage of shoots giving proliferative response, shoot morphology, length of main and axillary shoots, amount of obtained shoots and buds on explant, culture fresh and dry weight, and proliferation ratio (the average sum of buds and shoots obtained on a single explant) were established. The mean growth indexes of FW and DW were calculated as described earlier [19].

### 4.3. Qualitative and Quantitative Polyphenolic Compound Analysis

Firstly, lyophilized and micronized shoot samples (100 mg) from each treatment were extracted using 30 mL of chloroform to remove chlorophyll at 25 °C for 15 min. Following this, the shoots were extracted using 30 mL of 80% methanol in an ultrasonic bath (Techpan, Warsaw, Poland) at 40 °C for 15 min, followed by two additional extractions with 15 mL of the same solution. The combined extracts were then evaporated completely under reduced pressure and stored at 4 °C until further analysis.

The evaporated extracts were subjected to quantitative analyses using a UPLC-DAD-ESI-MS/MS system comprising a UPLC-300 RS apparatus (Dionex, Dreieich, Germany), with an AmaZon SL ion trap mass spectrometer and ESI interface (Bruker Daltonik GmbH, Bremen, Germany). A Zorbax SB-C18 column (Agilent, Santa Clara, CA, USA) was used to separate polyphenolic compounds, as described previously [19]. The polyphenolic compounds were identified by comparing their retention times, UV, and mass spectra with data from the literature.

Quantitative analysis was performed using an Agilent Technologies 1290 Infinity HPLC apparatus (Santa Clara, CA, USA) with DAD detector. Dry extracts were dissolved in 2 mL of 80% methanol, and then filtered using 0.22 μm nylon filters. The injection volume was set at 10 µL, as described previously [19]. Calibration was performed using authentic reference compounds of caffeic acid (CA), rosmarinic acid (RA), salvianolic acid A and salvianolic acid F (SAF) obtained from Chem Faces Biochemical Co., Ltd. (Wuhan, China). For polyphenolic compounds lacking commercially available standards, quantification was based on calibration curves of similar compounds: caffeic acid for CTA; rosmarinic acid for MRA, PLA, and RAH; salvianolic acid A for SAK, salvianolic acid F for SAF isomer I and II. Each analysis was repeated three times, and the findings are reported in mg/g DW. The total phenolic content was determined by summing the amounts of all the quantified polyphenols present in the sample.

### 4.4. Culture Duration

The shoot cultures were grown on MS medium supplemented with IAA 0.1 mg/L and BAP 1 mg/L. Detailed growth conditions are given in Section 4.2. Shoot multiplication ratio, morphology, length of main and axillary shoots, and dry and fresh weight were recorded after 30, 40, and 50 days (subculture 38–40). Secondary metabolite production was established after 30, 40, and 50 days of cultivation, as described in Section 4.3.

### 4.5. Statistical Analysis

Each experiment was repeated three times, and results are reported as means ± standard errors. The means were subjected to a one-way analysis of variance (ANOVA), followed by Tukey’s post hoc test. The statistical analysis was performed using STATISTICA 13.1 (StatsoftPolska, Kraków, Poland).

## 5. Conclusions

This study presents the first comprehensive report on the optimization of *S. atropatana* shoot cultures to maximize growth and bioactive compound production. Cytokinin supplementation significantly enhanced proliferation and biomass accumulation, with the best results achieved using 1 and 2 mg/L BAP. Phenolic profiling revealed RA as the dominant compound for all treatments, reaching a maximum of approximately 16 mg/g DW. With extended cultivation, *S. atropatana* shoot biomass accumulation increased, reaching a peak at day 50. However, the level of RA did not increase beyond day 40. Observable signs of culture deterioration were noted after this point, which could suggest that day 40 represents the optimal harvest time for maximizing RA yield while maintaining cost-efficiency. These findings highlight the critical role of developmental stage and culture conditions in regulating secondary metabolism and provide a basis for the biotechnological exploitation of *S. atropatana* as a sustainable source of RA and other phenolic compounds through optimized in vitro culture systems.

## Figures and Tables

**Table 1:** The proliferation response of *S. atropatana* culture with regard to the type and concentration of the tested cytokinin.

Cytokinin (mg/L)	% of Response	% of Shoots	% of Buds
BAP 0.5	100 ± 0.0	57.76 ± 7.06	42.24 ± 7.06
BAP 1	96.67 ± 5.77	48.35 ± 4.23	51.65 ± 4.23
BAP 2	100 ± 0.0	56.59 ± 9.76	43.41 ± 9.76
mTOP 0.5	93.33 ± 3.33	58.82 ± 8.37	41.18 ± 8.37
mTOP 1	100 ± 0.0	54.14 ± 8.45	45.86 ± 8.45
mTOP 2	96.67 ± 5.77	50.39 ± 9.19	49.61 ± 9.19
BPA 0.5	100 ± 0.0	54.84 ± 9.00	45.16 ± 9.00
BPA 1	96.67 ± 5.77	44.22 ± 6.90	55.78 ± 6.90
BPA 2	100 ± 0.0	46.77 ± 2.26	53.23 ± 2.26
rBAP 0.5	93.33 ± 6.67	63.13 ± 6.23	36.88 ± 6.23
rBAP 1	100 ± 0.0	59.86 ± 2.30	40.14 ± 2.30
rBAP 2	100 ± 0.0	49.01 ± 2.20	50.99 ± 2.20
Control	25 ± 2.89	44.44 ± 13.48	55.56 ± 13.48

**Table 2:** The phenolic compounds present in 80% methanolic extract of *S. atropatana* shoot culture. Masses were obtained by negative mode analysis.

Peak No.	Rt	**(M-H)^−^**	**Main Fragments**	Tentative Compound
1	14.9	297	135	Caffeoyl-threonic acid
2	19	179	135	Caffeic acid
3	25.2	357	313, 269, 203	Prolithospermic acid
4	32.2	521	223, 197, 179, 161	Rosmarinic acid hexoside
5	37.5	359	223, 197, 179, 161	Rosmarinic acid
6	38.1	555	537, 493, 359, 161	Salvianolic acid K
7	44.0	373	311, 197, 179, 135	Methyl rosmarinate
8	51.9	313	269, 161	Salvianolic acid F isomer I
9	53.9	313	269, 203, 161	Salvianolic acid F isomer II

**Table 3:** Performance score for the tested cytokinin treatments, calculated by TOPSIS.

Treatment	Performance Score
C	0.132
0.5 BAP	0.635
1 BAP	**0.782**
2 BAP	**0.773**
0.5 mTOP	0.347
1 mTOP	0.683
2 mTOP	0.569
0.5 BPA	0.260
1 BPA	0.600
2 BPA	0.744
0.5 rBAP	0.586
1 rBAP	0.456
2 rBAP	0.639

Bold—the highest performance scores.

## Data Availability

The data are contained within the article.

## References

[1] El Kantar S., Yassin A., Nehmeh B., Labaki L., Mitri S., Naser Aldine F., Hirko A., Caballero S., Monck E., Garcia-Maruniak A. (2022). Deciphering the therapeutical potentials of rosmarinic acid. Sci. Rep..

[2] Adomako-Bonsu A.G., Chan S.L.F., Pratten M., Fry J.R. (2017). Antioxidant activity of rosmarinic acid and its principal metabolites in chemical and cellular systems: Importance of physico-chemical characteristics. Toxicol. In Vitro.

[3] Cuvelier M.E. (2002). Antioxidative activity and phenolic composition of pilot-plant and commercial extracts of sage and rosemary. J. Am. Oil Chem. Soc..

[4] Andrade J.M., Faustino C., Garcia C., Ladeiras D., Reis C.P., Rijo P. (2018). *Rosmarinus officinalis* L.: An update review of its phytochemistry and biological activity. Future Sci. OA.

[5] Elansary H.O., Szopa A., Kubica P., Ekiert H., El-Ansary D.O., Al-Mana F.A., Mahmoud E.A. (2020). Saudi *Rosmarinus officinalis* and *Ocimum basilicum* L. polyphenols and biological activities. Processes.

[6] Bejenaru L.E., Biţă A., Mogoşanu G.D., Segneanu A.E., Radu A., Ciocîlteu M.V., Bejenaru C. (2024). Polyphenols investigation and antioxidant and anticholinesterase activities of *Rosmarinus officinalis* L. species from southwest Romania flora. Molecules.

[7] Espinosa-Leal C.A., Puente-Garza C.A., García-Lara S. (2018). In vitro plant tissue culture: Means for production of biologically active compounds. Planta.

[8] Muráriková A., Kaffková K., Raab S., Neugebauerová J. (2015). Evaluation of content of phenolics in *Salvia* species cultivated in South Moravian Region. Acta Fac. Pharm. Univ. Comen. LXII.

[9] Akdeniz M., Yener I., Dincel D., Firat M., Karatas Degirmenci D., Ertas A. (2022). Determination of fingerprints contents of different extracts and parts of six endemic *Salvia* taxa by GC–MS: Source species for valuable compounds with drug or drug potential. Biomed. Chromatogr..

[10] Areias F., Valentão P., Andrade P.B., Ferreres F., Seabra R.M. (2000). Flavonoids and phenolic acids of sage: Influence of some agricultural factors. J. Agric. Food Chem..

[11] Askari S.F., Avan R., Tayarani-Najaran Z., Sahebkar A., Eghbali S. (2021). Iranian Salvia species: A phytochemical and pharmacological update. Phytochemistry.

[12] Ertas A., Yigitkan S., Orhan I.E. (2023). A focused review on cognitive improvement by the genus *Salvia* L. (Sage)—From ethnopharmacology to clinical evidence. Pharmaceuticals.

[13] Radmanesh H., Tehranipour M., Sazgarnia A. (2021). Effect of aqueous extract of *Salvia atropatana* leaf on subcutaneous tumor model of CT26 colon carcinoma in mice. J. Gorgan Univ. Med. Sci..

[14] Abdollahi-Ghehi H., Sonboli A., Ebrahimi S., Esmaeili M., Mirjalili M. (2019). Triterpenic acid content and cytotoxicity of some *Salvia* species from Iran. Nat. Prod. Commun..

[15] Shakeri A., Farahmand S.S., Tayarani-Najaran Z., Emami S.A., Kúsz N., Hohmann J., Boozari M., Tavallaie F.Z., Asili J. (2021). 4,5-Seco-5,10-friedo-abietane-type diterpenoids with anticancer activity from *Salvia atropatana* Bunge. Naunyn-Schmiedeberg’s Arch. Pharmacol..

[16] Mirza M., Ahmadi L. (2000). Composition of the essential oil of *Salvia atropatana* Bunge. J. Essent. Oil Res..

[17] Fotovvat M., Radjabian T., Saboora A. (2019). HPLC fingerprint of important phenolic compounds in some *Salvia* L. species from Iran. Rec. Nat. Prod..

[18] Kharazian N. (2013). Identification of flavonoids in leaves of seven wild growing *Salvia* L. (*Lamiaceae*) species from Iran. Prog. Biol. Sci..

[19] Grzegorczyk-Karolak I., Ejsmont W., Kiss A.K., Tabaka P., Starbała W., Krzemińska M. (2024). Improvement of bioactive polyphenol accumulation in callus of *Salvia atropatana* Bunge. Molecules.

[20] Krzemińska M., Hnatuszko-Konka K., Weremczuk-Jeżyna I., Owczarek-Januszkiewicz A., Ejsmont W., Olszewska M.A., Grzegorczyk-Karolak I. (2023). Effect of light conditions on polyphenol production in transformed shoot culture of *Salvia bulleyana Diels*. Molecules.

[21] Attaran Dowom S., Abrishamchi P., Radjabian T., Salami S.A. (2017). Enhanced phenolic acids production in regenerated shoot cultures of *Salvia virgata* Jacq. after elicitation with Ag⁺ ions, methyl jasmonate and yeast extract. Ind. Crops Prod..

[22] Grzegorczyk-Karolak I., Hnatuszko-Konka K., Zarzycka M., Kuźma Ł. (2020). The stimulatory effect of purine-type cytokinins on proliferation and polyphenolic compound accumulation in shoot culture of *Salvia viridis*. Biomolecules.

[23] Hano C. (2022). Plant Hormones: Recent Advances, New Perspectives and Applications.

[24] Hallmark H.T., Rashotte A.M. (2019). Cytokinin response factors: Responding to more than cytokinin. Plant Sci..

[25] Rademacher W. (2015). Plant growth regulators: Backgrounds and uses in plant production. J. Plant Growth Regul..

[26] Pasternak T.P., Steinmacher D. (2024). Plant growth regulation in cell and tissue culture in vitro. Plants.

[27] Echeverrigaray S., Carrer R.P., Andrade L.B. (2010). Micropropagation of *Salvia guaranitica* Benth. through axillary shoot proliferation. Braz. Arch. Biol. Technol..

[28] Makunga N.P., Van Staden J. (2008). An efficient system for the production of clonal plantlets of the medicinally important aromatic plant: *Salvia africana-lutea* L.. Plant Cell Tissue Organ Cult..

[29] Misic D., Grubisic D., Konjevic R. (2006). Micropropagation of *Salvia brachyodon* through nodal explants. Biol. Plant.

[30] Koszeghi S., Bereczki C., Balog A., Benedek K. (2014). Comparing the effects of benzyladenine and meta-topolin on sweet basil (*Ocimum basilicum*) micropropagation. Not. Sci. Biol..

[31] Weremczuk-Jeżyna I., Skała E., Kuźma Ł., Kiss A.K., Grzegorczyk-Karolak I. (2019). The effect of purine-type cytokinin on the proliferation and production of phenolic compounds in transformed shoots of *Dracocephalum forrestii*. J. Biotechnol..

[32] Piątczak E., Owczarek A., Lisiecki P., Gonciarz W., Kozłowska W., Szemraj M., Chmiela M., Kiss A.K., Olszewska M.A., Grzegorczyk-Karolak I. (2021). Identification and quantification of phenolic compounds in *Salvia cadmica* Boiss. and their biological potential. Ind. Crops Prod..

[33] Grzegorczyk-Karolak I., Krzemińska M., Kiss A.K., Olszewska M.A., Owczarek A. (2020). Phytochemical profile and antioxidant activity of aerial and underground parts of *Salvia bulleyana* Diels. Metabolites.

[34] Zengin G., Llorent-Martínez E.J., Fernández-de Córdova M.L., Bahadori M.B., Mocan A., Locatelli M., Aktumsek A. (2018). Chemical composition and biological activities of extracts from three *Salvia* species: *S. blepharochlaena, S. euphratica var. leiocalycina*, and *S. verticillata* subsp. amasiaca. Ind. Crops Prod..

[35] Grzegorczyk-Karolak I., Krzemińska M., Kiss A.K., Owczarek-Januszkiewicz A., Olszewska M.A. (2023). Role of phytohormones in biomass and polyphenol accumulation in *Salvia bulleyana* in vitro culture. Biomolecules.

[36] Grzegorczyk-Karolak I., Kuźma Ł., Wysokińska H. (2017). The influence of cytokinins on proliferation and polyphenol accumulation in shoot cultures of *Scutellaria altissima* L.. Phytochem. Lett..

[37] Bekircan T., Yaşar A., Yıldırım S., Sökmen M., Sökmen A. (2018). Effect of cytokinins on in vitro multiplication, volatiles composition and rosmarinic acid content of *Thymus leucotrichus* Hal. shoots. 3 Biotech.

[38] Weremczuk-Jeżyna I., Kuźma Ł., Kiss A.K., Grzegorczyk-Karolak I. (2018). Effect of cytokinins on shoot proliferation and rosmarinic and salvianolic acid B production in shoot culture of *Dracocephalum forrestii*. Acta Physiol. Plant..

[39] Skała E., Wysokińska H. (2004). In vitro regeneration of *Salvia nemorosa* L. from shoot tips and leaf explants. In Vitro Cell. Dev. Biol. Plant.

[40] Tour J., Khalida K. (2014). In vitro regeneration of *Salvia santolinifolia*. Pak. J. Bot..

[41] Yadav A., Kothari S.L., Kachhwaha S., Joshi A. (2019). In vitro propagation of chia *(Salvia hispanica* L.) and assessment of genetic fidelity using random amplified polymorphic DNA and intersimple sequence repeat molecular markers. J. Appl. Biol. Biotechnol..

[42] Santos-Gomes P.C., Seabra R.M., Andrade P.B., Fernandes-Ferreira M. (2002). Phenolic antioxidant compounds produced by in vitro shoots of *Salvia officinalis* L.. Plant Sci..

[43] Bektaş E. (2020). Changes in essential oil composition, phenylalanine ammonia lyase gene expression and rosmarinic acid content during shoot organogenesis in cytokinin-treated *Satureja spicigera* (C. Koch) boiss. shoots. J. Plant Biochem. Biotechnol..

[44] Tyagi K., Maoz I., Kochanek B., Sela N., Lerno L., Ebeler S.E., Lichter A. (2021). Cytokinin but not gibberellin application had major impact on the phenylpropanoid pathway in grape. Hortic. Res..

[45] Andersen B.R., Jin G., Chen R., Ertl J.R., Chen C.M. (1996). Transcriptional regulation of hydroxypyruvate reductase gene expression by cytokinin in etiolated pumpkin cotyledons. Planta.

[46] Esmaeili H., Karami A., Maggi F. (2018). Essential oil composition, total phenolic and flavonoids contents, and antioxidant activity of *Oliveria decumbens* Vent. (*Apiaceae*) at different phenological stages. J. Clean. Prod..

[47] Hazrati S., Mollaei S., Rabbi Angourani H., Hosseini S.J., Sedaghat M., Nicola S. (2020). How do essential oil composition and phenolic acid profile of *Heracleum persicum* fluctuate at different phenological stages?. Food Sci. Nutr..

[48] Rao B.R., Bhattacharya A., Kaul P., Ramesh S. (2001). Yield and chemical composition of rose-scented geranium (*Pelargonium* species) oil at different times of harvesting. J. Essent. Oil Res..

[49] Hazrati S., Beidaghi P., Beyraghdar Kashkooli A., Hosseini S.J., Nicola S. (2022). Effect of harvesting time variations on essential oil yield and composition of *Salvia officinalis*. Horticulturae.

[50] Farhat M.B., Chaouch-Hamada R., Sotomayor J.A., Landoulsi A., Jordán M.J. (2014). Antioxidant potential of *Salvia officinalis* L. residues as affected by the harvesting time. Ind. Crops Prod..

[51] Sellami I.H., Maamouri E., Chahed T., Wannes W.A., Kchouk M.E., Marzouk B. (2009). Effect of growth stage on the content and composition of the essential oil and phenolic fraction of sweet marjoram (*Origanum majorana* L.). Ind. Crops Prod..

[52] Ayan A.K., Yanar P., Cirak C., Bilgener M. (2007). Morphogenetic and diurnal variation of total phenols in some *Hypericum* species from Turkey during their phenological cycles. Bangladesh J. Bot..

[53] Yang L., Wen K.S., Ruan X., Zhao Y.X., Wei F., Wang Q. (2018). Response of plant secondary metabolites to environmental factors. Molecules.

[54] Hosseini S.J., Tahmasebi-Sarvestani Z., Pirdashti H., Modarres-Sanavy S.A.M., Mokhtassi-Bidgoli A., Hazrati S., Nicola S. (2021). Investigation of yield, phytochemical composition, and photosynthetic pigments in different mint ecotypes under salinity stress. Food Sci. Nutr..

[55] Ilieva M., Pavlov A. (1997). Rosmarinic acid production by *Lavandula vera* MM cell-suspension culture. Appl. Microbiol. Biotechnol..

[56] Karam N.S., Jawad F.M., Arikat N.A., Shibl R.A. (2003). Growth and rosmarinic acid accumulation in callus, cell suspension, and root cultures of wild *Salvia fruticosa*. Plant Cell Tissue Organ Cult..

[57] Hakkim F.L., Kalyani S., Essa M., Girija S., Song H. (2011). Production of rosmarinic acid in *Ocimum sanctum* (L.) cell suspension cultures by the influence of growth regulators. Int. J. Biol. Med. Res..

[58] De-Eknamkul W., Ellis B.E. (1984). Rosmarinic acid production and growth characteristics of *Anchusa officinalis* cell suspension cultures. Planta Med..

[59] Lee S.Y., Xu H., Kim Y.K., Park S.U. (2008). Rosmarinic acid production in hairy root cultures of *Agastache rugosa* Kuntze. World J. Microbiol. Biotechnol..

[60] Roy D., Mukhopadhyay S. (2012). Enhanced rosmarinic acid production in cultured plants of two species of *Mentha*. Indian J. Exp. Biol..

[61] Moumou Y., Trotin F., Dubois J., Vasseur J., El-Boustani E. (1992). Influence of culture conditions on polyphenol production by *Fagopyrum esculentum* tissue cultures. J. Nat. Prod..

[62] Murashige T., Skoog F. (1962). A revised medium for rapid growth and bio assays with tobacco tissue cultures. Physiol. Plant..

